# Current Trends in the Monitoring and Treatment of Diabetic Retinopathy in Young Adults

**DOI:** 10.1155/2014/492926

**Published:** 2014-02-13

**Authors:** Dorota Raczyńska, Katarzyna Zorena, Beata Urban, Dominik Zalewski, Andrzej Skorek, Grażyna Malukiewicz, Bartosz L. Sikorski

**Affiliations:** ^1^Department of Anesthesiology and Intensive Care Medicine, Department of Ophthalmology, Medical University of Gdańsk, Mariana Smoluchowskiego 17, 80-214 Gdańsk, Poland; ^2^Department of Clinical and Experimental Endocrinology, Institute of Maritime and Tropical Medicine, Medical University of Gdańsk, Powstania Styczniowego 9b, 81-519 Gdynia, Poland; ^3^Department of Pediatric Ophthalmology and Strabismus, Medical University of Bialystok, Waszyngtona 17, 15-274 Bialystok, Poland; ^4^Diagnostic and Microsurgery Center of the Eye Lens, Budowlana 3A, 10-424 Olsztyn, Poland; ^5^Department of Otolaryngology, Medical University of Gdańsk, Dębinki 7, 80-952 Gdańsk, Poland; ^6^Department of Ophthalmology, Nicolaus Copernicus University, M. Sklodowskiej-Curie 9, 85-090 Bydgoszcz, Poland

## Abstract

The diagnosis and treatment of diabetic retinopathy (DR) in young adults have significantly improved in recent years. Research methods have widened significantly, for example, by introducing spectral optical tomography of the eye. Invasive diagnostics, for example, fluorescein angiography, are done less frequently. The early introduction of an insulin pump to improve the administration of insulin is likely to delay the development of diabetic retinopathy, which is particularly important for young patients with type 1 diabetes mellitus (T1DM). The first years of diabetes occurring during childhood and youth are the most appropriate to introduce proper therapeutic intervention before any irreversible changes in the eyes appear. The treatment of DR includes increased metabolic control, laserotherapy, pharmacological treatment (antiangiogenic and anti-inflammatory treatment, enzymatic vitreolysis, and intravitreal injections), and surgery. This paper summarizes the up-to-date developments in the diagnostics and treatment of DR. In the literature search, authors used online databases, PubMed, and clinitrials.gov and browsed through individual ophthalmology journals, books, and leading pharmaceutical company websites.

## 1. Introduction

Diabetes mellitus (DM) is a major health problem worldwide. Current studies have revealed a definite global increase in the incidence and prevalence of diabetes, with the World Health Organization (WHO) projecting that there will be up to 285 million cases in the year 2025 [1]. Although this increase is mainly expected in type 2 diabetes (T2DM), a parallel increase in childhood diabetes, including T1DM and T2DM, has been reported [2].

The main concern in diabetes is the development of changes connected with micro- and macrocirculation. In the course of diabetic microangiopathy, changes that are clinically most important occur within the small vessels of the retina, the kidneys, and the nervous system [3]. Macroangiopathy concerns the coronary artery disease, cerebral stroke, and peripheral artery disease. The changes occur in medium and large arteries [4].

## 2. Epidemiology of Diabetic Retinopathy (DR)

Diabetic retinopathy (DR) is a microvascular complication of diabetes and one of the common causes of visual impairment and loss of working days in middle-aged adults, while cataract and refractive errors are still the leading cause of blindness in children [5]. DR estimates gradually declined between 1990 and 2004 and then dramatically declined in 2010: it was a cause of 4,8% of global blindness in 2002, 3,9% in 2004 and 1% in 2010 [6]. The report from United States describes the results of diabetic patients analysis, which indicated that although the number of adults with diagnosed diabetes reporting visual impairment increased, the percentage of adults with diagnosed diabetes, who reported visual impairment, declined significantly, from 23.7% in 1997 to 16.7% in 2010 [7]. The growth of diabetes and DR is a major concern for developing countries [8]. In addition, there is a high proportion of undiagnosed diabetes in developing countries.

## 3. Prevalence of DR in Young Adults

The 20-year analysis of 1,604 adolescents with T1DM has shown that the prevalence of retinopathy has continued to decrease, resulting from better glycemic control [9]. Authors observed that retinopathy was found in approximately 50% of adolescents with T1DM after a median duration of 9 years in the early 1990s, compared with only 12% in recent years.

Nowadays, lower-than-expected prevalence and severity of DR can be noticed. Lecaire et al. in 2006 observed less severe DR than expected, with a very low prevalence of moderate-severe nonproliferative retinopathy (10%) and only one person was treated of PDR by 14 years' duration [10]. In contrast, in 1984 at the baseline evaluation in WESDR, moderate-severe nonproliferative retinopathy was found in 35% of persons and PDR in 25% of persons at 13-14 years' duration of diabetes [11]. Likewise, Skrivarhaug et al. in 2006 proved low cumulative incidence of PDR in childhood-onset T1DM during a 24-year follow-up study [12]. Nine out of every ten patients diagnosed with T1DM developed DR, but only one out of ten developed PDR within their first 25 years of diabetes duration, so the cumulative incidence of PDR is lower than previously reported from other countries. Similar tendency can be observed in the prevalence of diabetic macular edema (DME). The Visconsin Epidemiologic Study of Diabetic Retinopathy showed a reduction in the incidence of DME in the last period of the 25-year observation of diabetic patients. A reason for the decline in the incidence of DME may reflect recent improvement in diabetes care and better glycemic control [13].

## 4. Risk Factors for DR

### 4.1. Duration of Diabetes

Many studies demonstrated that the determining factor for the development of vascular complications and ocular changes is the duration of diabetes [11, 14–17]. 30 years ago in Wisconsin Epidemiologic Study of Diabetic Retinopathy, 996 young patients with T1DM were examined [11]. Within the examined population after 5 or less years of T1DM duration in 17% features of DR were determined and in the group with over 15 years of diabetes duration as much as 97.5%. In another research Klein et al. demonstrated that, among the 271 examined patients with T1DM diagnosed before the age of 30 without symptoms of DR in the beginning of this study, after 4 years of diabetes duration as much as 59% of patients have developed nonproliferative DR as well as in 11% of patients frequency of PDR increased to 14% after 13 years of diabetes duration. Furthermore, DR deterioration occurred in 41% of patients, whereas improvement in visual acuity was observed only in 7% of diabetic patients [17]. Simsek et al., while examining 1,032 patients from 12 different centers in Turkey, detected presence of DR in 1,4% young patients with T1DM. In examined patients, the presence of DR was correlated with the age of patients and longer duration of diabetes [14]. In the patients that we examined, who suffered from diabetes for over 6 years, there was a large risk of DR and diabetic nephropathy [18]. However, later in the research we have determined that it was not for the duration of diabetes but for the tumor necrosis factor alpha (TNF-*α*) that the highest discriminant value was demonstrated in predicting the development of microangiopathy in youth with T1DM.

### 4.2. Genetic Predispositions

The results of the research suggest that the genetic predisposition for the development of DR is connected with the presence of HLA DR3/DR4 antigens. Furthermore, what was demonstrated is the influence of different allelic forms of cytokine-, chemokine-, and growth factor coding gene on its functional properties on the level of transcription, as well as the relation with DR in patients with T1DM and T2DM. From a diabetologist's and ophthalmologist's perspective, discovering the existence of polymorphic places in genes coding VEGF and TGF*β* cytokines, the aldose reductase gene, paraoxonase (PON1), and the nitrous oxide synthase gene was essential [19–25]. An interesting discovery was made by Ray et al., who analyzed the occurrence of C/G polymorphism in the promoter region of the VEGF gene in −460 position [24]. The authors demonstrated that allele C more frequently appears in patients with PDR rather than nephropathy. Data from other studies also point to the role of TGF-*β*1 gene polymorphism in the pathogenesis of DR. Beránek et al. have confirmed a more frequent occurrence of 915G/C (R25P) polymorphism in patients with DR compared to control subjects [25]. At this time, no definite genetic associations with DR have been consistently reported.

### 4.3. Arterial Hypertension

According to the current guidelines from the *International Society for Pediatric and Adolescent Diabetes (ISPAD)*, young patients with T1DM should have their arterial blood pressure measured at least once a year [26]. According to the guidelines from the National High Blood Pressure Education Program blood pressure measurement should be an essential part of every visit to the pediatrician and/or diabetologist, similarly to full physical examination, the evaluation of HbA1c concentration, or microalbuminuria [27]. In recent studies it has been demonstrated that an insufficient blood pressure decrease at night and an increased number of improper levels of arterial pressure are connected with an increased risk of diabetic complications [28–31]. In the study by Gallego et al., it has been demonstrated that both systolic and diastolic pressure may be a prognostic factor for DR [31]. Furthermore, the same authors have shown that blood pressure increases the probability of earlier retinopathy regardless of incipient nephropathy in younger patients with T1DM [31].

### 4.4. Obesity in Children and Youth

The childhood obesity epidemic has become one of the most challenging problems of the modern society, mostly for its clinical and social consequences [32, 33]. Body mass index (BMI = weight in kg/height in m²) is the most widely used parameter to assess obesity. Adults are considered obese if BMI ≥ 30 kg/m². BMI changes with age in children, and therefore absolute cut offs are not appropriate for them. Instead, childhood obesity is defined as BMI ≥ 95th percentile, respectively, as per age and gender-specific BMI references. The pattern of distribution of body fat is a better determinant of morbidity than BMI alone. Central (visceral or abdominal) fat deposition is associated with a higher risk of cardiovascular disease and diabetes mellitus, in comparison to gluteal or subcutaneous fat. Waist circumference is generally used as a measurement of central obesity [34]. In most cases obesity in the developmental age later develops into adulthood obesity and with its prolonged duration arterial hypertension, atherosclerosis, ischaemic heart disease, and retinopathy begin to develop [14, 29, 35, 36]. Similarly to adults, it has been demonstrated that in children a strict relationship between obesity and arterial hypertension as well as DR occurs [25, 29, 36]. Simultaneously, with the increased frequency of obesity in children and adolescents an increased frequency of disturbances in carbohydrate metabolism can be observed [35]. Most often biochemical disturbances occur, which may precede the presence of T2DM; these include insulin resistance accompanied by hyperinsulinism and improper blood glucose curve in oral glucose tolerance test (OGTT). In the research done by Sinha et al. [35], impaired glucose tolerance was noted in 21% of obese children aged 11–18 and in 25% of obese children aged 4–10. Patients with overweight or with obesity are subject to increased risk of suffering from dyslipidemia. Lipid disturbances occur in 12–17% of children with overweight. In the studies by Jago et al., it has been determined that in children with overweight and obesity low concentration of HDL cholesterol and higher level of triglycerides are noted significantly more frequently [28]. It is worth noting that obesity is observable not only in children and adolescents with T2DM but is also present in children and adolescents with T1DM [14].

### 4.5. Disturbances of Lipid Metabolism

**Figure 1:**
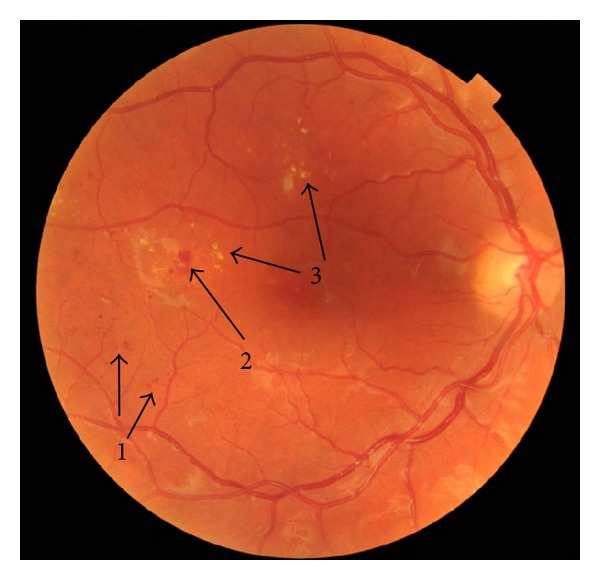
Image of 28-year-old patient with T1DM and DR (1: microaneurysms, 2: hemorrhages, and 3: hard exudates).

The symptom of lipid disturbances during prolonged DR is the deposition of lipids i. lipoproteins in the retina, especially in the macula, and the formation of “hard exudates” (Figure 1). These appear on the fundus of the eye in the form of dots, spots, or plaques. The deposition of “hard exudates” in the macula is the main element in the process of the formation of diabetic maculopathy [37, 38]. In the study by Minuto et al., conducted on 247 young patients with T1DM, retinopathy was found in 26/247 patients. A significant relationship between retinopathy and serum triglycerides levels >65 mg/dL was found [37]. In another research a pilot study was conducted among 222 individuals with T1DM and 43 with T2DM who participated in the SEARCH for Diabetes in Youth study. The prevalence of DR was 17% for T1DM and 42% for T2DM. LDL cholesterol was also significantly higher among those with any DR compared with those without DR. This pilot study suggests that, despite advances in diabetes care, DR remains an important concern both in terms of research and clinical care. The authors suggest that further long-term study of DR in youth is needed [38].

### 4.6. Pregnancy

Diabetic retinopathy can worsen during pregnancy because of the pregnancy itself or due to changes in metabolic control [8, 39]. Patients with diabetes who are planning to become pregnant should be encouraged to have their eyes examined prior to conception; they should be counseled on the risk of development and/or progression of DR and should be told to make every attempt to lower their blood glucose levels as close to normal as possible for their own health and the health of the fetus [39]. During the first trimester, another eye examination should be performed; subsequent followup will depend on the determined level of retinopathy [8]. Women who develop gestational diabetes do not require an eye examination during pregnancy, because such individuals are not at increased risk for DR during pregnancy.

### 4.7. Puberty

In the development of DR more importance is being given to puberty in young patients with T1DM. One of the first researches suggested that puberty has no significant effect on the development of DR [40]. However, since then, many publications appeared in which the authors determine that puberty indeed contributes to the development of late diabetic complications [14, 41–43]. Comparative studies demonstrate that the development and progression of DR are faster in the year after puberty. In a detailed analysis, a slower effect of the development of DR was determined in patients under 5 years of age, in which the risk of DR increased every year before puberty and after it. In current studies a relationship between puberty and the frequency as well as the severity of DR was observed. Furthermore it has been demonstrated that higher frequency of DR occurs after puberty than before it, regardless of the diabetes duration and metabolic control [41, 42]. Likewise Harvey in his recent claim determined that patients after puberty have a 3,2 times greater risk of retinopathy than patients who were before their puberty [43]. In recent studies conducted on a large group of children with T1DM it has been determined that DR was present in 1.9% of pubertal patients and in 0.3% of prepubertal patients [14].

### 4.8. Smoking Cigarettes

Smoking is a well-known risk factor for cardiovascular disease in both diabetic and nondiabetic persons. However, the effects of smoking on DR are unclear. Some studies have suggested an association, while others have not [44–46]. In T2DM smoking cigarettes may protect against the progression of retinopathy in some patients, despite the fact that it is an independent risk factor for myocardial infarction and death from cardiovascular disease in patients with diabetes [45]. Mühlhauser has reviewed this problem and concluded that association between smoking and DR remains less consistent than the association between smoking and nephropathy [46]. Nevertheless, smoking in young patients with DM should be discouraged.

## 5. Pathogenesis of DR

Both experimental and clinical studies showed that an essential role in the pathogenesis of chronic diabetic complications is played by hyperglycemia. Most publications note that the damaging action among cells takes place by activating some metabolic pathways, whereby the nonenzymatic protein glycation is considered to be the most important.

### 5.1. Advanced Glycation Endproducts (AGEs)

The presence of advanced glycation endproduct is closely related to hyperglycaemia and its pathobiochemistry could explain many of the changes observed in DR [47]. AGEs accumulation in the walls of vessels leads to their damage. Glication of collagen in basement membrane of retinal capillaries induces its progressive thickening, vascular lumen narrowing with loss of elasticity, hypertension, and endothelial dysfunction. Several AGEs receptors have been identified and RAGE is the best characterized AGE receptor. It is located on endothelial cells, macrophages and microglia [48]. AGE-RAGE binding on these cells leads to oxidant stress and activation of the transcription factor NF-*κ*B. NF-*κ*B modulates gene transcription for endothelin-1, VCAM-1, tissue factor and thrombomodulin [49]. It also regulates release of IL-1*α*, IL-6, and TNF-*α*. In vitro studies have shown that AGE modification of retinal basement membrane results in loss of pericytes, increased permeability, and increased retinal endothelial cell proliferation [47]. Other important features of AGE-induced vasculopathy include effect on coagulation and fibrinolysis, leading to occlusion and ischaemia as well as induction of growth factors such as VEGF, resulting in angiogenesis [47].

Many studies indicate that pathophysiological mechanisms leading to the vascular complications of diabetes include (1) endothelial dysfunction, (2) an activation of the inflammation cascade, and (3) procoagulant imbalance [50]. Their circulating biomarkers may therefore provide opportunities for early diagnosis and targets for novel treatment of DR. Circulating biomarkers of these pathways such as TNF-*α*, IL-6, C-reactive protein (CRP) (inflammation), vascular cellular adhesion molecule-1, interstitial cellular adhesion molecule-1, E-selectin, von Willebrand factor (endothelial dysfunction), plasminogen activator inhibitor-1, fibrinogen, P-selectin (procoagulant state), and adiponectin (antiinflammation) may be associated with development of both type 1 and type 2 diabetes complications [50]. Several research groups, including ours, demonstrated that certain biomarkers may have independent predictive value. Similarly studies have shown that these biomarkers may be associated with development of DR [51, 52].

### 5.2. Endothelial Dysfunction: VCAM-1, ICAM-1, E-Selectin, and von Willebrand Factor

Endothelial dysfunction plays an important role in the development of DR. The term endothelial dysfunction refers to an impairment of the ability of the endothelium to properly maintain vascular homeostasis, and it may be an important determinant of altered vascular reactivity. The most critical mediator of endothelium-derived molecules is nitric oxide (NO) and the earliest and most important marker of endothelial dysfunction is represented by a reduction in NO bioactivity [53]. Many biochemical pathways associated with hyperglycemia can increase the production of free radicals by reducing the amount of biologically active NO. The activation of protein kinase C, nicotinamide-adenine dinucleotide phosphate depletion, and the formation of AGEs in diabetes cause a decrease of NO and vascular dysfunction. These events could lead to retinal vascular endothelial dysfunction and result in increased retinal blood flow and retinal vasodilation. Indeed, an abnormal retinal vascular response to hyperoxia is associated with the development of DR [54]. Clinical studies confirmed that endothelial dysfunction is common in children and adolescents with type 1 diabetes of short duration [55]. The measurement of the serum levels of endothelium-derived cellular adhesion molecules (CAMs), such as endothelin-1 (ET-1), intercellular adhesion molecule (ICAM-1), vascular cell adhesion molecule (VCAM-1), E-selectin and von Willebrand factor, and endothelium-dependent vasodilation, in addition to widely used indirect methods are employed to estimate the degree of endothelial dysfunction [56]. ICAM-1, VCAM-1, and ET-1 are important markers of endothelial dysfunction that have been demonstrated to play important roles in the development of DRP. ICAM-1 and VCAM-1 mediate leukocyte adhesion to the retinal vasculature, one of the earliest pathological changes in PDR [57]. Leukocyte adhesion to the retinal vasculature is one of the earliest pathological changes observed in the development of DR and leads to enhanced vascular permeability, endothelial cell damage and capillary nonperfusion [50, 57]. The ICAM-1 level has been shown to be increased in the diabetic retina, even in the early stages of retinopathy. Moreover, the administration of neutralizing anti-ICAM-1 antibodies causes a dramatic reduction in the incidence of leukocyte-related pathologies in newly diabetic animals [57]. ET-1 is one of the most potent vasoconstrictor molecules causing abnormalities in retinal hemodynamics, thereby contributing to the development of PDR [58]. Increased ICAM-1, VCAM-1, and E-selectin are associated with nephropathy, retinopathy, and cardiovascular disease in both T1DM and T2DM [50]. von Willebrand factor (vWF) is a marker for endothelial dysfunction and mediates platelet adhesion. Increased vWF was associated with a prolonged retinal circulation time and reduced retinal blood flow in early-stage retinopathy of type 1 diabetes [59]. Reduced blood flow associated with increased vWF levels may promote stasis in the retinal circulation and lead to local hypoxemia. These changes might contribute to the microvascular complications of diabetes [60].

### 5.3. An Activation of the Inflammation Cascade

DR is recognized as a chronic low-grade inflammatory disease. Circulating biomarkers of inflammation include TNF-*α*, IL-6, and CRP. Diabetes causes metabolic and physiologic abnormalities in the retina, and these changes suggest a role for inflammation in the development of DR. These changes include upregulation of iNOS, COX-2, ICAM-1, caspase 1, VEGF, and NF-kappaB, increased production of nitric oxide, prostaglandin E2, IL-1beta, and cytokines, and increased permeability and leukostasis [61]. Diabetic subjects have an overall increased inflammatory activity compared to nondiabetic subjects, as demonstrated by increased serum levels of TNF-*α* [62]. Serum TNF-*α* concentrations over 1.7 pg/mL may point to the presence of diabetic microangiopathy in children and adolescents with type 1 diabetes [18]. TNF-*α* and IL-1*β* attenuated the migration and capillary morphogenesis of retinal endothelial cells. These dysfunctions were associated with an increased production of reactive oxygen species, expression of inducible NO synthase, and production of total nitrate/nitrite. Incubation of retinal EC with TNF-*α* and IL-1*β* altered VE-cadherin localization, as well as the expression of other junctional proteins. In addition, TNF-*α* and IL-1*β* also altered the production of various ECM proteins including osteopontin, collagen IV, and tenascin-C. These changes were concomitant with the activation of the mitogen-activated protein kinase (MAPK) and nuclear factor-*κ*B (NF-*κ*B) signaling pathways [63]. IL-1beta accelerates apoptosis of retinal capillary cells via activation of NF-kappaB, and the process is exacerbated in high glucose conditions. These studies suggest a possible role of IL-1beta in the development of retinopathy in diabetes and offer a possible rationale to test IL-1beta receptor antagonists to inhibit the development of DR [64].

### 5.4. Procoagulant Imbalance

Researchers demonstrated a variety of mechanisms contributing to the thrombotic tendency in patients with DM, such as increased platelet aggregation and adhesion, increased fibrinogen production, abnormal levels of clothing factors (VII, VIII, XI, XII, kallikrein, and von Willebrand), decreased fibrinolytic activity (through low levels of t-PA and high levels of PAI-1), and increased blood viscosity [47, 50, 65]. Decreased levels of antithrombin III and thrombomodulin were noted in hyperglycemia [66]. These plasma proteins are potent anticoagulants inhibiting clot formation. Asakawa et al. observed significantly higher levels of fibrynogen in diabetic patients who had DR and nephropathy [67].

### 5.5. Adiponectin

Adiponectin represents an adipocyte-specific secretory protein modulating endothelial cell functions. Adiponectin has been found to have antiinflammatory, antiatherogenic, and cardioprotective properties. Morales et al. noted that adiponectin levels in pediatric type 1 diabetic subjects did not differ from those of healthy control subjects [68]. On the contrary, Celi et al. observed that adiponectin concentrations were higher only in the prepubertal diabetic children [69]. Many studies have shown higher adiponectin levels in patients with T1DM compared with patients with T2DM [52]. Zietz et al. observed that elevated adiponectin serum levels are associated with DR in patients with T2DM [70]. The adiponectin concentration was also significantly higher in patients with T1DM and severe DR than in those without retinopathy [71].

## 6. Screening of DR

### 6.1. Screening of Preclinical DR in Children and Adolescents

DR is microvascular complication; however, the retina is primarily neural tissue. Kurtenbach et al. have demonstrated neuroretinal dysfunction, including delayed multifocal oscillatory potentials in patients with diabetes before the appearance of vascular lesions [72]. Similarly, standard multifocal electroretinogram (mfERG) studies have shown delayed implicit times in patients with diabetes that are exacerbated in patients with nonproliferative DR [73]. In patients with nonproliferative DR, localized retinal areas with delayed mfERG timing have been shown to precede the development of new vascular lesions [74]. Multifocal ERG showed an increase in areas of localized neuroretinal dysfunction in adolescents with type 1 diabetes and no clinically visible DR [75]. Neuroretinal dysfunction in patients without clinically detectable retinopathy can be also detected in blue-on-yellow perimetry and in contrast sensitivity [76, 77]. Findings from these studies suggest that measures of localized neuroretinal function could be useful in detection of the early changes associated with DR in young diabetic adults.

### 6.2. Screening for Diabetic Retinopathy in Children and Adolescents

For children with T1DM, the majority of guidelines recommend first examination to commence at or soon after puberty [8]. In paediatric DR, annual screening is recommended by many ophthalmologists, with mydriatic stereoscopic fundus photography being the most sensitive detection method [78]. Current guidelines recommend annual retinopathy screening 2 years after onset (for pubertal-onset type 1 diabetes) and after 5 years (or age 11, whichever is earlier) for prepubertal onset [79]. American Diabetes Association and American Academy of Pediatrics guidelines recommend annual eye exams for children with T1DM who are older than 10 years, starting 3–5 years after diagnosis [80]. As the rate of T2DM in children is increasing, the number of children with complications of diabetes, such as DR, will also increase.

## 7. Detection of DR

Early detection of DR is extremely important in the prevention of visual impairment and in monitoring ocular complications, especially in young patients with T1DM [81].

The current standard methods to screen DR are the best correct vision acuity (BCVA) examination, slit lamp biomicroscopy, dilated fundus examination with ophthalmoscope, intraocular pressure measurement on patients with glaucoma risk, and most importantly digital fundus color photos graded by trained image graders and fluorescein angiography (FA) [8].

### 7.1. Retinal Photography

Retinal photography has been reported to be the most sensitive screening method for DR. Ophthalmoscopy has less sensitivity but conversely a higher specificity. It provides good results in the hands of trained professionals such as ophthalmologists and diabetologists, especially when used in repeated examinations [82]. Retinal photography for DR has been promoted for decades for both the screening of the disease and in landmark clinical research studies, such as the Early Treatment Diabetic Retinopathy Study (ETDRS) [83]. Stereophotography is more reliable for detecting an increase in retinal thickness, but rather laborious and time consuming [78]. Systematic review of evidence suggests that mydriatic photography is the most effective screening strategy, with high sensitivity (87–97%) and specifity (83–92%) for detection of sight-threatening DR, but it has several disadvantages (time taken to obtain and interpret the photographs, the need for dilating drops and its associated issues related to patient compliance) [8].

### 7.2. Nonmydriatic Photography

The limitations of mydriatic photography prompted experts to propose the use of nonmydriatic retinal cameras for DR screening. Nonmydriatic digital stereoscopic retinal imaging is a sensitive and specific method for the screening and diagnosis of DR, which may help improve compliance with the standards of eye care for patients with diabetes [84]. The nonmydriatic image capture with a scanning laser ophthalmoscope provides the additional benefits of easier operation, no pupil dilation, and more rapid acquisition. The limitations of nonmydriatic photography for DR screening are noticed in the literature. It should be noted that when retinal cameras are used without mydriasis, the technical failure rates may be as high as 20–36% [85]. Silva et al. compared nonmydriatic stereoscopic Optomap ultrawide field images with dilated stereoscopic Early Treatment Diabetic Retinopathy Study 7-standard field 35-mm color 30-degree fundus photographs (ETDRS photography) [86]. There were 14 eyes, in which Optomap images did not identify PDR seen on ETDRS photographs. Authors conclude that the excellent photographic image quality is needed to identify subtle neovascularization that otherwise can be obscured easily without sharp focus, optimal illumination, high contrast, and good color balance.

### 7.3. Fluorescein Angiography (FA)

The major advantage of FA over fundus photography is its ability to detect macular ischemia denoted by nonperfusion of the retinal capillaries and to detect subtle DME as evidenced by fluorescein leakage from the capillaries [87]. Drawbacks to using FA as a screening procedure are its invasiveness, time constraints, expensive equipment, and adverse reactions. Allergic-type reactions have been reported in patients undergoing FA, although the incidence of serious complications is rare. In general, the use of FA is limited to determining method and location of laser photocoagulation for DME and for assessing the extent of nonperfusion. It has limited value over photography as a diagnostic tool and is not recommended for routine use. It is not needed to diagnose clinically significant ME or PDR, both of which are diagnosed by means of the clinical examination [8]. The prolonged followup of diabetic retinopathy in childhood demonstrated that the early changes are not necessarily a negative prognostic factor in the evolution of DR and early FA is not particularly useful in the management of children with diabetes [88].

### 7.4. OCT, RTA, and HRT

**Figure 2:**
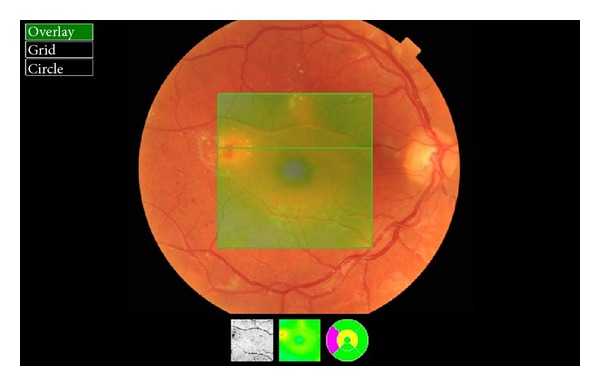
Retinal thickness map—patient from Figure 1.

**Figure 3:**
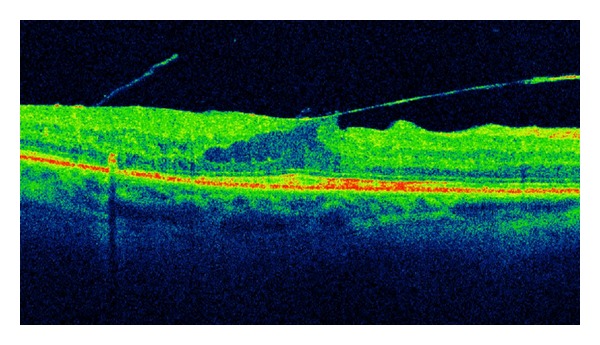
SD-OCT of the patient with T1DM and vitreomacular traction syndrome.

The optical coherence tomography (OCT) is a relatively new test. OCT is a device that has revolutionized the diagnosis of eye diseases. Imaging of ocular tissues in section comparable to histologic pictures is an invaluable diagnostic tool in the study of both the anterior and posterior segments of the eye [89]. With OCT it is possible to assess DME and vitreoretinal tractions (Figures 2 and 3). OCT is also easy to perform in young patients. It does not require mydriasis, takes only a few minutes, and the result is available immediately after the test. The advantage of the diagnostic effectiveness of OCT in macular edema over standard fundus ophthalmoscopy was rated by Hee et al. [90]. Studying DME, an increase in measure of 50% or more central macular thickness was observed by OCT, which had been overlooked during the standard fundus examination.

In the evaluation of macular thickness, measurement precision was significantly higher for the OCT in comparison to the retinal thickness analyzer (RTA) in virtually all areas of the retina. A significant increase in retinal thicknesses in eyes with macular edema was observed in all areas by the OCT, but only for average foveal thickness by the RTA [91]. Another method capable for evaluating the macular edema is Retina Module of the Heidelberg retina tomograph II (HRTII), which not only detects morphological changes, but also relates to functional changes [92].

## 8. Treatment of DR

The treatment of DR includes increased metabolic control, laserotherapy, farmacological treatment (antiangiogenic and anti-inflammatory treatment, enzymatic vitreolysis, and intravitreal injections), and surgery.

### 8.1. Increased Metabolic Control

Primary interventions such as intensive glycemic control, strict blood pressure regulation, and lipid-modifying therapy can significantly reduce the risk of retinopathy occurrence and progression [93]. Close cooperation between the diabetologist and the ophthalmologist is crucial for the success of diabetic complications treatment.

Continuous subcutaneous insulin infusion (CSII), often called insulin pump therapy, was introduced in the 1970s as a way of achieving and maintaining strict control of blood glucose concentrations in people T1DM. The use of insulin pump can effectively improve the mean glucose values and reduce the percentage of HbA1C and therefore reduces or delays DR. Insulin pumps appear to offer potential benefit over multiple daily injections (MDI) [94]. Although several studies demonstrated a reduced risk of microvascular complications in adults and adolescents treated with intensive management, the association between intensive treatment regimens (CSII or MDI) and improved glycemic control is less clear in children [94, 95]. Furthermore, there is no evidence demonstrating a reduced risk of complications in children treated with CSII or MDI versus 1 to 2 injections per day. There are very few studies demonstrating a specific benefit of CSII over MDI on complications in adolescents. Downie et al. proved that there was a reduced risk of DR in adolescents with type 1 diabetes treated with CSII versus MDI [9]. In the study of 1,604 adolescents with type 1 diabetes, the prevalence of DR has continued to decrease in parallel with an intensification of management. Charles et al. showed that improved glycemic control from CSII led to a lower incidence of diabetes complications, with the most significant reduction in PDR [96].

### 8.2. Laserotherapy

Currently, laser photocoagulation is the primary method of treatment for patients with diabetic retinopathy who are at a high risk of vision loss. The aim of retinal laser photocoagulation is (1) destroying the retina in areas of vascular hypoperfusion, (2) coagulation of vascular abnormalities, (3) reduction of macular edema, and (4) better “fixation” of the retina to the choroid [97].

Among the methods of laserotherapy in DR we can distinguish (1) focal photocoagulation, (2) diffuse (scatter) photocoagulation, and (3) panretinal photocoagulation [98, 99]. Focal photocoagulation is a popular method of complementary laserotherapy at every stage of DR. Diffuse laserotherapy is used in preproliferative DR and in diffuse DME [100]. Panretinal photocoagulation is performed only in patients with PDR [99, 101]. ETDRS report number 19 showed that laser photocoagulation significantly reduces severe vision impairment in eyes at high risk [98]. Unfortunately it is not always effective for improving vision. In many cases laser treatment can simply maintain vision and avoid further vision loss. It is established that in about 50% of patients retinopathy progresses despite laser photocoagulation. The procedure is uncomfortable, and often repeated treatments are required. Moreover, laser photocoagulation is an ablative, retinal tissue destroying procedure, where scars always enlarge over time leading to decrease in night vision, colour vision, and peripheral vision as well as loss of 1 or 2 lines of visual acuity in some patients [102].

### 8.3. Anti-VEGF Antibodies, VEGF Inhibitors

**Figure 4:**
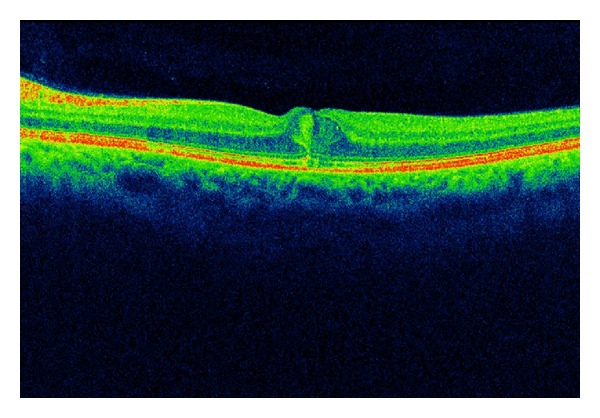
SD-OCT of the 29-year-old patient with T2DM and DME.

**Figure 5:**
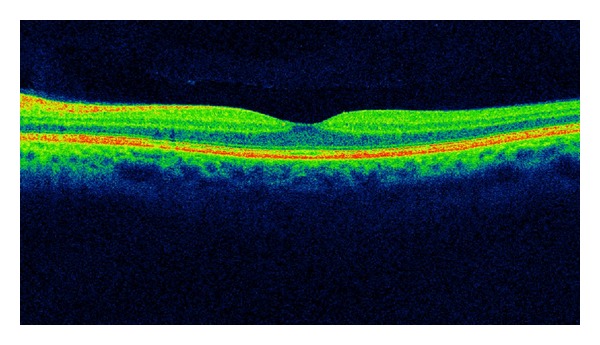
Regression of DME after one anti-VEGF intraocular injection—patient from Figure 4.

Because VEGF is the key link in stimulating angiogenesis, blocking it produces effective results and inhibition of VEGF has become popular method of the treatment of DR [103]. VEGF is a key mediator of angiogenesis and the substance responsible for the interruption of the blood-retina barrier in patients with DR. Unfortunately, the effect of anti-VEGF is transient and requires reinjection. Equally good results are also obtained by treatment with intravitreal administration of ranibizumab (Lucentis.) and bevacizumab (Avastin) (Figures 4 and 5). Bevacizumab is a complete full-length humanized antibody that binds to all subtypes of VEGF and is successfully used in tumor therapy as a systemic drug [104]. Ranibizumab is a recombinant humanized antibody fragment against VEGF-A. It is the first angiogenesis inhibitor approved by the FDA for the treatment of DME [105]. According to a recent study comparing the efficacy of both drugs in the treatment of DME, bevacizumab and ranibizumab were associated with similar effects on the central subfield thickness in patients with DME through 1 year of followup. Ranibizumab is associated with greater improvement in BCVA, while the mean number of injections is higher in the bevacizumab group [104]. The conclusions of the multicenter study of ranibizumab in the treatment of DME are promising: after use of ranibizumab in 40–60% of patients with DME, visual acuity improved by 2 lines in 23–32% of patients [106]. Ranibizumab-treated eyes with DME were 2-3 times less susceptible to loss of visual acuity as compared with the laser monotherapy [107].

The latest intravitreal drug used in DME is an aflibercept known as VEGF Trap-Eye (Eylea). It is a recombinant fusion protein consisting of portions of human VEGF receptors 1 and 2 extracellular domains fused to the Fc portion of human IgG1. This VEGF inhibitor has the ability to bind all isoforms of VEGF and placental growth factor (PlGF) [108]. In phase II of the da Vinci Study in patients with DME both visual acuity improvement and good tolerance to the drug were observed [109].

### 8.4. Corticosteroids (Intravitreal Injections, Implants)

Since inflammation is identified as a relevant mechanism in the development of DR, significant effort has been directed to the development of new concepts for the prevention and treatment of DR acting on the inflammatory processes and the use of pharmacological agents with anti-inflammatory effect. Due to this fact, intravitreal and subconjunctival injections or intraocular implants of corticosteroids bring good effects. These drugs have the ability to inhibit VEGF and other proinflammatory factors (e.g., IL-6, IL-8, MCP-1, IP-10, and IFN-*γ*) [110]. In addition, corticosteroids can block all the various steps involved in leukostasis, including downregulating the selectins and integrins [111]. Their spectrum of activity is wider than that of anti-VEGF. As shown in animal tests anti-VEGF drugs do not block, for example, the proinflammatory effects of IL-1*β* and TNF-*α* [112]. Major adverse effects of intravitreal corticosteroids include the induction or worsening of cataracts and elevated intraocular pressure [112, 113]. It is worth noting that due to the administration of the drugs directly into the eye systemic corticosteroid side effects are avoided. Triamcinolone acetate is administered intravitreally either “off label” or as registered Triesence preparation (40 mg/mL, Alcon) and Trivaris (80 mg/mL, Allergan). As numerous studies have shown, it improves visual acuity, inhibits neovascularization, and delays development of the disease [114].

*Implants.* Implants are either longer-acting and nonresorbable (fluocinolone acetonide implants) or shorter-acting and resorbable (dexamethasone implants). At this time, no steroid is approved by the United States Food and Drug Administration (FDA) for the treatment of DME.

At present, there are several implants available.

*Retisert* (Bausch & Lomb) is an intraocular nondecomposing biological implant. The active substance releases fluocinolone acetate up to 3 years at a dose of 0.5 *μ*g/day. Three years of research in patients with DME showed improvement in visual acuity and the lack of progress of DR [115]. During the test the efficacy of Retisert in DME patients showed the total disappearance of diabetic macular edema after implantation in 53.7% of patients compared with 28.6% in patients who did not receive corticosteroids.

*I-vation* (SurModics, Inc.) is a helix with a length of 5 mm releasing triamcinolone acetonide and implanted into the eye. Phase I of clinical trials has been completed. According to the manufacturer the possible duration of the spiral can be from 1 month to 3 years. The spiral can serve as carrier for various other drugs for DR therapy [116].

*Iluvien* (Alimera Sciences) is an injectable, nonerodible, fluocinolone acetonide implant that is approved in several European countries for the treatment of DMO. It can be injected into the back of the eye and is biodegradable. Each implant provides a therapeutic effect of up to 36 months [117].

*Ozurdex* (Oculex Pharmaceuticals Inc., Allergan) is an extended-release biodegradable dexamethasone intravitreal implant. The drug is in the form of a stick. In DME clinical trials using Ozurdex, improvement in visual acuity of 15 letters was observed in 18.1% of patients receiving a dose of 700 *μ*g, as opposed to 5.7% of patients in the control group [118].

The* Verisome* delivery system (Icon Biosciences, Inc.) is a sustained-release drug delivery system. IBI-20089 is an intraocular sustained release product for the delivery of triamcinolone acetonide. It is administered as a standard intravitreal injection. Formulated as a gel, IBI-20089 forms a sphere in the posterior segment after intravitreal injection. This sphere gradually degrades and disappears as the drug is released in a controlled manner [119].

The* Cortiject implant* (NOVA63035; Novagali Pharma) is a preservative and solvent-free emulsion that contains a proprietary tissue-activated corticosteroid prodrug. As a prodrug activated at the retinal level, it is intended to efficiently treat macular edema. A single intravitreal injection of the emulsion provides sustained release of the corticosteroid over a 6–9-month period. An open-label, phase I, dose-escalation clinical study to assess the safety and tolerability of NOVA63035 in patients with diabetic retinopathy is currently ongoing [120].

### 8.5. Drugs Involved in Enzymatic Vitreolysis

Formation of epiretinal membranes (ERM) and vitreoretinal tractions are often observed in patients with diabetes. They arise as a result of cell proliferation on the surface of the retina and by the deposition of material probably produced by cells. The epiretinal membranes together with the accompanying vitreoretinal tractions are usually the reason for the formation of a macular hole, macular edema, and retinal detachment [121]. In case of the presence of proliferative lesions, it is possible to use enzymatic vitreolysis (vitreous body liquefaction). Among the interchangeable enzymes are, for example, recombinant tissue plasminogen activator (rTPA), hyaluronidase, urokinase, plasmin, ocriplasmin, dyspase, chondroitinase ABC, and collagenase. The first enzyme approved by the FDA in 2012 for the treatment of vitreoretinal traction is ocriplasmin (Yetrea, ThromboGenics). It is a recombinant protease with activity against fibronectin and laminin and components of the vitreoretinal interface. The MIVI-TRUST trials have shown that intravitreal injection of ocriplasmin was superior to a placebo injection in a dissolution of the vitreoretinal traction [122].

### 8.6. Surgery

Surgical treatment in severe cases may include both the anterior and posterior segment of the eye. Within the anterior segment it may be necessary to remove cataract and to perform antiglaucoma surgery. Surgery in the posterior segment of the eye—posterior vitrectomy—is recommended in the cases of nonabsorbent hemorrhage into the vitreous cavity, PDR, with, for example, tractional retinal detachment, proliferation of vitreoretinal membranes (PVR), epiretinal membrane formation, and vitreoretinal traction syndrome. To obtain a positive effect during vitrectomy, the vitreous base is removed, which is a “scaffold” for proliferating membranes and new vessels [123]. Vitrectomy combined with the administration of triamcinolone acetonide and laser therapy has a beneficial effect on both anatomical and functional outcomes in DME resistant to anti-VEGF [124].

Posterior vitrectomy in advanced cases of PDR is a costly procedure, difficult, and burdened with high risk. Patients prepared for this type of operation should be informed of the prognosis and the possible need to reoperate in the future. However, in advanced stages of PDR, surgery remains the only form of treatment.

## 9. Summary

Care of young adults with diabetes should include an interdisciplinary approach of a diabetologist, a pediatrician, and an ophthalmologist. The patient should be given continuity of treatment and remain under monitoring. This is particularly important in T1DM, where the probability of faster development of DR is higher. Despite the many new drugs and surgical techniques, it seems crucial to maintain blood sugar at the right level. An insulin pump may delay the development of diabetic retinopathy and decrease the need for an intervention in the eye. Surgical intervention should be undertaken with caution due to the tendency for an increased proinflammatory response in the eyes of children and young adults. In the case of retinopathy, because of the complex etiology of diabetes, the combination therapy using a variety of techniques and drugs may be the most effective.
